# Supplementary material for: A study on altruistic consumption of Chinese residents from the perspective of intergenerational income mobility
Source: PLoS One. 2024 Jan 29;19(1):e0297444. doi: 10.1371/journal.pone.0297444 (PMC10824438; doi:10.1371/journal.pone.0297444)
Supplement: Attachment — Submitted filename: Review PONE-D-23-19313.docx [file pone.0297444.s001.docx]

Review for PONE-D-23-19313

This paper analyzes the theoretical mechanism through which intergenerational income mobility affects residents' marginal propensity to consume and altruistic consumption.

In the field of intergenerational income mobility research, a number of literature reviews have been analyzed for measuring the level of intergenerational income mobility.

The authors perform an analysis based on the OLG model. Also, in the paper, IGE is used to measure the power of the level of intergenerational income mobility. Based on the measurement of IGE and MPC, the authors build a model to analyze the effect of Intergenerational Income Elasticity on residents' Marginal Propensity to Consume using provincial panel data.

The results of the Hausman test required simple processing that led to the validation of the hypotheses assumed by the authors.

In the Introduction section, the values of several studies are highlighted, but the identified reference sources are not mentioned.

The bibliography does not follow alphabetical order.

I believe that the work can be published after making a minor revision.

Thanks to the editors for the opportunity to review this paper.
