# Supplementary material for: A study on altruistic consumption of Chinese residents from the perspective of intergenerational income mobility
Source: PLoS One. 2024 Jan 29;19(1):e0297444. doi: 10.1371/journal.pone.0297444 (PMC10824438; doi:10.1371/journal.pone.0297444)
Supplement: Attachment — Submitted filename: Response to Reviewers.docx [file pone.0297444.s002.docx]

First, I would like to thank the academic editors and reviewers for suggesting changes to my manuscript. The following responses are made to each point raised by the academic editor and reviewers.

1) The research in this paper is promoted based on the Diamond’s Overlapping Generation model. However, the Overlapping Generation model was not introduced in the previous manuscript, resulting in the Related Literature section of the paper was incomplete. In the revised manuscript, we have added the OLG model in the Related Literature section. The cited references have also been added.

2) The division standard of youth, middle aged, and old aged residents have been explained in the article. Youth are defined as those who are not yet able to work and are in the education stage. Middle aged residents are defined as those who are able to earn income from labor and need to raise children. And old aged residents are defined as those who are not able to work and therefore cannot continue to earn income from labor.

3) The reviewers noted that the meanings of some notations have not been well explained such as *a*_0_, *a*_1_,*δ*_i_ in Eqs. (12) and (13). In the revised manuscript, we have made additions.

4) “In the last decade, about 7%-9% of the income composition of China's residents is property income, and over 70% is wage and business income.” The data involved in the above sentence were calculated by the author on the basis of official data published by the National Bureau of Statistics of China. The sources of the data have been explained in the revised manuscript.

5) In the Analysis of Urban-Rural Heterogeneity section, we introduced the interaction term of Intergenerational Income Elasticity (IGE) and urban-rural classification (urban) into the model, and then estimate the model with MPC_1_ and MPC_2_ as explanatory variables, respectively. The results show that the interaction term has a positive effect on MPC_1_ and MPC_2_ at the 1% level of significance. We therefore conclude that: compared with rural residents, Intergenerational Income Elasticity has a stronger positive effect on urban residents' marginal propensity to consume。

6) In the revised manuscript, we have explained the reference sources of the relevant studies and data in the Introduction section. The results of the Hausmann test are also shown in the revised manuscript.

7) The References in the original manuscript were sorted according to the order of citation. Based on the reviewers' suggestions, we reordered the bibliography in alphabetical order in the revised manuscript.
