# Supplementary material for: A study on altruistic consumption of Chinese residents from the perspective of intergenerational income mobility
Source: PLoS One. 2024 Jan 29;19(1):e0297444. doi: 10.1371/journal.pone.0297444 (PMC10824438; doi:10.1371/journal.pone.0297444)
Supplement: Attachment — Submitted filename: Review PONE-D-23-19313 v2.docx [file pone.0297444.s003.docx]

Review for PONE-D-23-19313

This paper analyzes the theoretical mechanism through which intergenerational income mobility affects residents' marginal propensity to consume and altruistic consumption.

The authors considered the comments of the reviewers. However, there are still some aspects that can lead to a better form of paper.

1. Keyword review is required: Marginal propensity to consume / Intergenerational income mobility / Altruistic motivation.
2. In the Introduction section, the authors still start with a numerical presentation without indicating the source. An appropriate wording would be ... According to the source, in 2014, China's total final consumption …
3. At the end of the introduction section, the structure of the work by sections is not presented.
4. In Related Literature Section, Heading 2 are required for *Diamond’s Overlapping Generation model (2.1)* and *Literature on Intergenerational Income Mobility (2.2).* Also Heading 3 for Section 4.1 and 5.2 (4.1.1. … ; 5.2.1 ….). Use the same format for headings.
5. In explanation, when you refer to the table columns use "and" (2)(4) use (2) and (4). I also found an error in the paragraph about two words about the column: The results indicate that the fixed-effects model should be chosen, so we use the estimation results in column columns (2)(4) …

I believe that the work can be published after making a minor revision.

Thanks to the editors for the opportunity to review this paper.
