# Supplementary material for: A study on altruistic consumption of Chinese residents from the perspective of intergenerational income mobility
Source: PLoS One. 2024 Jan 29;19(1):e0297444. doi: 10.1371/journal.pone.0297444 (PMC10824438; doi:10.1371/journal.pone.0297444)
Supplement: Attachment — Submitted filename: Response to Reviewers.docx [file pone.0297444.s004.docx]

First, I would like to thank the academic editors and reviewers for suggesting changes to my manuscript. The following responses are made to each point raised by the academic editor and reviewers.

1) According to the reviewer's suggestion, I did the sensitivity evaluation of the model in order to show prediction performance of the proposed model. The results of the analysis are presented after the benchmark results. I have also added a description of the age division standard in the paper.

2) I did a review of the keywords and revised them.

3) In the Introduction section,. the sources of the data are indicated. At the end of the introduction section, I present the structure of the paper by sections.

4) The serial numbers of the article section headings have been added and adjusted. Word errors raised by reviewers have also been corrected.

Thanks again to the academic editors and reviewers for their comments on this paper!
